# Sudden Death in Brazil: Epilepsy Should be in Horizon

**DOI:** 10.5935/abc.20150072

**Published:** 2015-08

**Authors:** Fulvio Scorza, Paulo José Ferreira Tucci

**Affiliations:** Escola Paulista de Medicina - Unifesp, São Paulo, SP - Brazil

**Keywords:** Death, Sudden / prevention & control, Death, Sudden, Cardiac, Coronary Artery Disease / mortality, Epilepsy / mortality

To date, a considerable amount of valuable information about the problem of sudden cardiac death (SCD) has been described. The incidence of SCD in the United States ranges between 180000-400000 cases per year^1^. Martinelli et al demonstrated an incidence of 21270 cases of SCD per year in the Metropolitan Area of São Paulo^2^. Recently, Braggion-Santos et al.^3^ described the characteristics of SCD in Ribeirão Preto, Brazil, according to autopsy reports^3^. Revising 4501 autopsies, they identified 899 cases of SCD (20%); the rate was 30/100000 residents/year^3^. The vast majority of SCD cases involved coronary artery disease (64%). Based on available scientific knowledge related to SCD, it is extremely important to identify new areas of research that might improve understanding of this problem and to establish effective preventive measures to minimize or even control the occurrence of SCD.

Although studies have shown that the increase in the number of SCD caused by a combination of factors^2,3^, an equally important risk factor for SCD which is not reported and not explored in cardiologic research is epilepsy. Indeed, a series of data could be put forward to explain it.

Epilepsy affects approximately 65 million individuals worldwide and is one of the most common, chronic and severe neurological diseases^4-7^. In developing and poor countries, the incidence of epilepsy is higher when compared with that of developed countries^4-7^. The prognostic evolution has clearly shown that seizures are successfully controlled with currently available antiepileptic drugs in approximately two-thirds of individuals with epilepsy, which results in one-third with refractory epilepsy^4,8^. For these patients with uncontrolled seizures, epilepsy should be considered a malignant condition, as it carries a mortality rate that is 2‑3 times higher than that in the general population^9^. Therefore, sudden unexpected death in epilepsy (SUDEP) is the most frequent cause of epilepsy-related death^9-12^. By definition, SUDEP is a sudden, unexpected, witnessed or unwitnessed, non‑traumatic and non-drowning death in individuals with epilepsy, with or without evidence of seizures, in which post-mortem examination does not reveal a toxicological or anatomical cause of death^13^. Epidemiological studies indicate that SUDEP is responsible for 7.5% to 17% of all deaths in epilepsy and has an incidence among adults between 1:500 and 1:1000 patient/year^14^. The main risk factors for SUDEP include the number of generalized tonic-clonic seizures, nocturnal seizures, young age at epilepsy onset, longer duration of epilepsy, dementia, absence of cerebrovascular disease, asthma, male gender, symptomatic etiology of epilepsy and alcohol abuse^12,15^. The cause or causes of SUDEP are still unknown, but one of the main proposed mechanisms is related to autonomic dysregulation, promoting cardiac abnormalities during and between seizures^16-18^.

In this line of reasoning, our experimental data clarified some possibilities. Using the pilocarpine model of temporal lobe epilepsy, we evaluated heart rate in rats with epilepsy* in vivo* and in an isolated *ex vivo* preparation (Langendorff preparation)^17^. Baseline heart rate *in vivo* in animals with chronic epilepsy (346 ± 7 bpm) was higher than in control rats (307 ± 9 bpm)^17^. Incidentally, no difference was observed in the isolated *ex vivo* situation (control animals: 175 ± 7 bpm; chronic epilepsy: 176 ± 6 bpm), suggesting that autonomic modulation of the heart is altered in epileptic animals, explaining the maintenance of an increased basal heart rate in these animals^17^. In addition, we also evaluated heart rate responses during stage 5 of amygdala kindling model, the phase when animals develop generalized seizures^18,19^. Animals did not show significant differences in basal heart rate; however, basal heart rate was higher during stage 5 of kindling, possibly resulting from sympathetic activation caused by the chronic epileptic condition^18,19^. As demonstrated in previous studies^20^, intense bradycardia at the beginning of seizure was followed by rebound tachycardia^18,19^. Moreover, the intensity of tachycardia was directly related to the number of generalized seizures, suggesting that repeated generalized tonic-clonic seizures affect sympathetic outflow^18,19^. For that reason, a plausible explanation is that continuous and intermittent sympathetic activation due to uncontrolled seizures is capable of maintaining cardiac rhythm, modulating the heart in accelerated-state permanently.

Considering all these translational information, it is clear that epilepsy-related mortality, particularly SCD, is a significant public health concern. Thus, it is crucial that a concerted and collaborative approach be implemented to solve this problem. In order to do so, it is extremely necessary to attain a real convergence between cardiologists and neurologists to carefully evaluate and discuss the electroencephalographic and electrocardiographic recordings, the cardiac and cerebral imaging findings and refined histopathological studies in order to detect or prevent the occurrence of a tragic fatal event among individuals with epilepsy.
